# Radiation Recall Dermatitis in Patients Treated With Immune Checkpoint Inhibitors: A Case Report and Literature Review

**DOI:** 10.7759/cureus.15548

**Published:** 2021-06-09

**Authors:** Ecem Yigit, Deniz Can Guven, Sercan Aksoy, Gozde Yazici

**Affiliations:** 1 Radiation Oncology, Hacettepe University Medical School, Ankara, TUR; 2 Medical Oncology, Hacettepe University Medical School, Ankara, TUR

**Keywords:** immunotherapy, immune checkpoint inhibitors, nivolumab, radiation recall, radiation recall dermatitis, radiotherapy

## Abstract

Radiation recall dermatitis (RRD) is defined as a skin reaction in the previously irradiated area triggered by a systemic agent's administration. The use of immune checkpoint inhibitors (ICI) alone and in combination with other treatments is increasing in many cancers. ICI-associated radiation recall reactions such as dermatitis, pneumonia, and myelitis have been reported so far. We report a case of nivolumab (anti-programmed cell death protein-1 antibody) induced RRD in a patient with head and neck cancer and review the publications reporting RRD associated with other ICI in the literature. The patient was diagnosed with neck metastasis of unknown primary origin and underwent surgery followed by adjuvant chemoradiotherapy (CRT). During the follow-up, radiotherapy (RT) was performed to the left parotid region, right neck level 1b, and the left neck skin due to recurrence. After three months of the last RT session, she was started on nivolumab due to the metastatic disease. Four weeks later, she was represented with erythematous squamous plaque-like lesions starting from the left temporomandibular region and spreading to the anterior chest, which corresponded to the previously irradiated area. A biopsy was performed with the differential diagnosis of skin metastases which revealed subacute spongiotic dermatitis. The lesions completely regressed in two weeks with the use of topical steroids and antihistamine tablets. Nivolumab treatment was not interrupted, and no reaction was observed during or after the next cycle. Although RRD is rarely encountered clinically, it is a diagnosis that should be kept in mind while continuing treatment with systemic agents in patients with a history of RT. With the widespread use of ICI, RRD associated with these treatments could be better defined and appropriately managed.

## Introduction

Radiation recall dermatitis (RRD) is defined as a skin reaction in the previously irradiated area triggered by a systemic agent's administration. D'Angio et al. [1] first described this phenomenon in 1959 induced by actinomycin-D. Cases of RRD induced by conventional chemotherapeutics, antibiotics, statins, tamoxifen, ultraviolet light exposure, amlodipine, hypericin, coronavirus disease 19 (COVID-19) vaccine, targeted therapies, and immunotherapies have been reported so far [2-6].

Targeted therapy (TT) could be defined as small synthetic molecules or monoclonal antibodies that act by blocking the action of specific enzymes, proteins, or other molecules involved in cancer cells' growth or by helping the immune system kill cancer cells. Immune checkpoint inhibitors (ICI) also help T cells kill cancer cells by preventing checkpoint proteins from binding to their common proteins. The use of TT and ICI alone and in combination with other treatments is increasing in cancers such as skin cancer, lung cancer, breast cancer, renal cell cancer (RCC), colorectal cancer, head and neck cancer, hepatocellular cancer (HCC), and lymphomas. Although they appear to be well-tolerated, different side effects may occur related to treatment.

ICI-associated radiation recall reactions such as dermatitis, pneumonia [7], and myelitis [8] have also been reported. We report a case occurring RRD after nivolumab (anti-programmed cell death protein-1 antibody) and review the publications reporting RRD associated with ICI in the literature. For the literature review, the keywords 'radiation recall', 'radiation recall dermatitis', 'RRD' were searched on MEDLINE. Cases over the age of 18 and publications in English were evaluated. There was no year limitation. The references used in the reviewed articles were also taken into consideration.

## Case presentation

**Figure 1:**
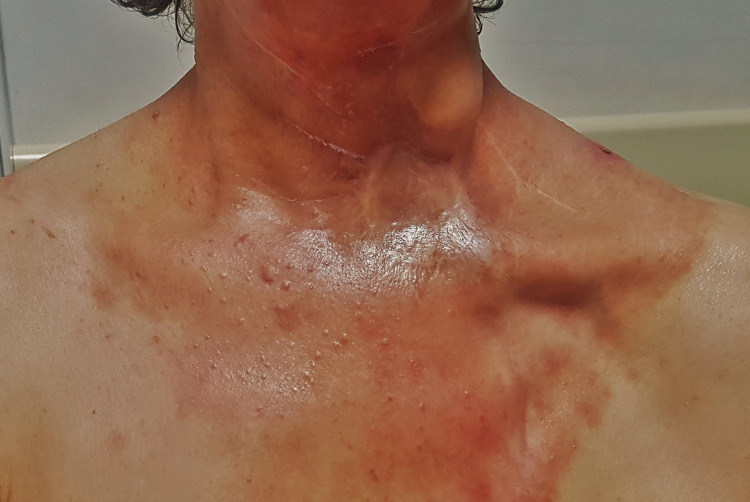
Erythematous plaque-like lesions after nivolumab administration.

**Figure 2:**
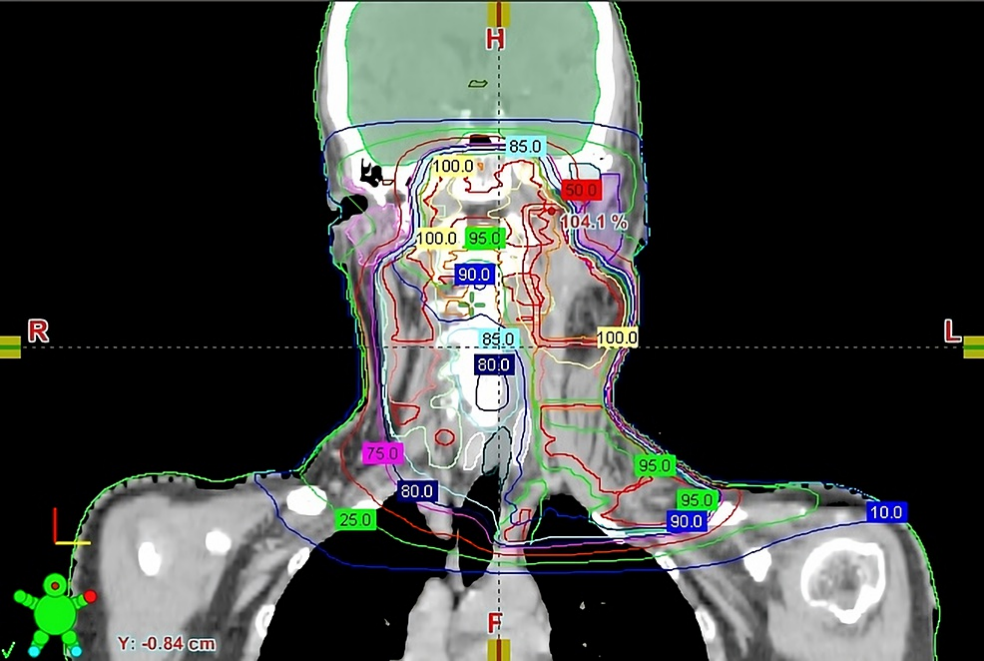
Coronal view of dosimetry showing RT isodoses (the outer blue line represents 10% isodose, corresponding to 640 cGy). RT: radiotherapy.

A 61-year-old female patient was diagnosed in August 2018 with neck metastasis of unknown primary origin. She underwent left radical neck dissection and was staged as pT0N2b squamous cell carcinoma (SCC) with sarcomatoid differentiation (according to American Joint Committee on Cancer (AJCC) 8th edition). She was treated by adjuvant concomitant cisplatin and radiation therapy (64 Gy: left neck level 1b-2, 60 Gy: tumor bed and left neck level 3, 57 Gy: left neck level 4-5, 54 Gy: right neck level 2-3-4 in 32 fractions with 6 MV photons using the intensity-modulated radiation therapy (IMRT) technique). During follow-up in December 2018, a recurrence was detected in the left parotid region outside the RT field. She underwent left parotidectomy, and the surgical margins were positive. She received adjuvant RT to the left parotid bed to a total dose of 40 Gy in 5 fractions with 6 MV photons using the IMRT technique. In May 2019, MRI showed an enlarged lymph node at the right neck level 1b, which was not previously irradiated, and the biopsy confirmed metastatic disease. Thereupon, 60 Gy RT was applied in 30 fractions with 6 MV photons using the volumetric arc treatment (VMAT) technique to the right neck level 1b with concomitant carboplatin. While the patient was followed up without medication, in January 2020, a 2.5 cm diameter erythematous soft tissue lesion developed on the left neck lateral skin. Excisional biopsy was performed, and pathologic examination revealed sarcomatoid carcinoma metastasis with a 0.6 cm surgical margin. 40 Gy RT in 5 fractions with 6 MV photons using the VMAT technique was performed to the tumor bed at the left neck skin. After three months of the last RT session, she was started on nivolumab due to the metastatic disease at the retrosternal lymph node and right lung. Four weeks later, erythematous plaque-like lesions were formed starting from the bilateral temporomandibular region and spreading to the anterior chest, which corresponded to the previously irradiated area (Figures 1, 2). A biopsy was performed with the differential diagnosis of skin metastases which revealed subacute spongiotic dermatitis. The lesions completely regressed in two weeks using topical steroids (hydrocortisone butyrate %0.1, twice daily) and antihistamine tablets (cetirizine dihydrochloride, once daily). Thus, nivolumab treatment was not interrupted, and no reaction was observed during or after the next cycle.

## Discussion

We presented a case of RRD that developed four weeks after nivolumab administration. Due to the increasing use of ICI, many case reports have been published in recent years regarding RRD induced by these treatments. Although the underlying mechanism is not precisely defined, there are different hypotheses such as epithelial stem cell inadequacy, vascular reactions, or drug hypersensitivity reaction after radiotherapy [9,10].

RRD may also be considered a late form of radiosensitization. Camidge et al. [10] suggested that if the skin reaction developed within the first seven days after RT, it should be evaluated as radiosensitization. RRD may develop within weeks or even years after RT, and the most extended period reported is 66 years [11]. Levy et al. [4] reported the median time to develop radiation recall with TT initiation as 19.6 weeks. Radiosensitization is a more common clinical entity compared to RRD. Increased radiosensitization has been reported with many TT and ICI. Horii et al. [12] reported pembrolizumab-associated Stevens-Johnson syndrome (SJS) as radiation recall dermatitis in a patient with malignant melanoma. In this case, pembrolizumab was initiated before RT and continued concurrently with RT. Therefore, it may be more appropriate to describe the case as radiosensitization. Nakashima et al. [13] described RRD in a non-small cell lung cancer (NSCLC) case treated with atezolizumab; however, they did not specify the interval between RT and drug.

If the triggering agent is started during RT or within the first week after RT, the skin reaction that develops is considered radiation enhancement [9]. Classically, if the agent was started after a safe time from RT, it is appropriate to call the reaction RRD. ICI-related cases reported in the literature are summarized in Table 1. The cases in which the triggering agent was started during RT or within seven days and the skin reaction developed within the first seven days after RT was omitted.

**Table 1:** ICI-induced RRD cases in the literature. d: days; fx: fractions; Gy: Gray; IV: intravenous; mg: milligram; m: months; NS: not specified; NSCLC: non-small cell lung cancer; PD-1: programmed cell death protein 1; PDL-1: programmed death-ligand 1, RT: radiation therapy; SCC: squamous cell carcinoma; SCLC: small cell lung carcinoma; w: weeks; ICI: immune checkpoint inhibitors; RRD: radiation recall dermatitis.

Drug	Effect mechanism	Dose	Primary histology	RT field, dose/fx	Time interval between RT and drug	Time to onset after drug	Treatment	Drug withheld	Rechallenge	Associated drugs	Study
Cemiplimab	PD-1 inhibitor	350 mg IV infusion	Cutaneous SCC	Left lower eyelid 66 Gy/30 Fx	12 w	≈4 w	Topical steroid, emollient cream, nicotinamide, antihistamine tablets	Yes	Yes	No	Vaccaro, 2020 [14]
Nivolumab	PD-1 inhibitor	NS	Melanoma	Left pelvis 9 Gy/3 Fx	1 w	A few hours	Topical steroid	Yes	Yes	No	Korman, 2017 [15]
			NSCLC	Thoracic 66 Gy, Left knee 30 Gy, Lumbar vertebrae 20 Gy/5 Fx	2 w	3 w	NS	NS	NS	No	Rouyer, 2018 [16]
			Breast carcinoma	Chest wall, 50 Gy/25 Fx	5 w	1 w	Topical steroid	Yes	Yes (Not tolerated)	No	Billena, 2020 [5]
			Melanoma	NS, 60 Gy/30 Fx	44.2 m	3.9 w	No	No	-	No	Deutsch, 2021 [17]
			Melanoma	Femur, 20 Gy/5 Fx	1.5 m	3 w	Steroid	Yes	No	Ipilimumab	
			Head and neck SCC	NS, 66 Gy/55 Fx	27.2 m	110 w	No	No	-	Lirilumab	
Pembrolizumab	PD-1 inhibitor	Oral 200 mg daily	SCLC	Primary lung lesion and lymphatics 50 Gy/20 Fx	6 m	3 d	Systemic steroid	NS	NS	NS	Wang, 2020 [18]

RRD may range from mild erythema and dry desquamation to skin necrosis and ulceration. There is no correlation between the total radiation dose and the severity of the skin reaction, yet it has been suggested that the skin reaction is more severe when the interval between KT and RT is shorter [1]. Rouyer et al. [16] reported a case in which nivolumab-induced RRD presenting as Stevens-Johnson's Syndrome.

There is no specific radiotherapy dose threshold for the development of RRD. In the case of RRD induced by docetaxel, Yeo et al. [19] observed that dermatitis did not occur in the region receiving a dose of 16.8 Gy, yet occur in the region receiving a dose of 18.7 Gy, and suggested that the threshold dose could be between 16.8-18.7 Gy. On the other hand, Stelzer et al. [20] treated different skin areas with a total dose of 40 Gy, 20 Gy, and 8 Gy with the diagnosis of Kaposi's sarcoma and reported that RRD developed only in the area treated with 40 Gy after bleomycin administration. Scher et al. [21] reported that dermatitis induced by applying a cream containing essential oils and natural plant extracts occurs in areas with a dose above 20 Gy on the skin, since cetuximab is also applied to the patient, it may affect the threshold dose value. Recently Billena et al. [5] observed nivolumab-induced RRD in the area treated with 50 Gy yet not in areas treated with 20 Gy. However, nivolumab-induced RRD was also reported at doses as low as 9 Gy [15].

There is no guideline for treatment, still, RRD mostly regresses spontaneously or with symptomatic treatment (topical/systemic steroids, antihistamines, non-steroidal anti-inflammatory drugs, emollient creams). Depending on the severity of dermatitis, the triggering agent can be discontinued. After skin reactions regressed, re-challenge was successful in most ICI-induced RRD cases. Re-challenge resulted in a more severe reaction requiring discontinuation of the drug in a patient diagnosed with breast cancer who developed nivolumab-associated RRD [5].

## Conclusions

Although radiation recall dermatitis is rarely encountered clinically, it is a diagnosis that should be kept in mind while continuing treatment with systemic agents in patients with a history of radiotherapy. With the widespread use of ICI, RRD associated with these treatments could be better defined and appropriately managed. As there are no specific risk factors and an established standard treatment approach for RRD, further studies are needed.
